# FAM122A, a new endogenous inhibitor of protein phosphatase 2A

**DOI:** 10.18632/oncotarget.11698

**Published:** 2016-08-30

**Authors:** Li Fan, Man-Hua Liu, Meng Guo, Chuan-Xi Hu, Zhao-Wen Yan, Jing Chen, Guo-Qiang Chen, Ying Huang

**Affiliations:** ^1^ Department of Pathophysiology, Key Laboratory of Cell Differentiation and Apoptosis of Chinese Ministry of Education, Rui-Jin Hospital, Shanghai Jiao Tong University School of Medicine, Shanghai 200025, China; ^2^ Institute of Health Sciences, Shanghai Institutes for Biological Sciences of Chinese Academy of Sciences and Shanghai Jiao Tong University School of Medicine, Shanghai 200025, China

**Keywords:** FAM122A, PP2A, ubiquitination, phosphatase activity, cell growth

## Abstract

The regulation of the ubiquitously expressed protein phosphatase 2A (PP2A) is essential for various cellular functions such as cell proliferation, transformation, and fate determination. In this study, we demonstrate that the highly conserved protein in mammals, designated FAM122A, directly interacts with PP2A-Aα and B55α rather than B56α subunits, and inhibits the phosphatase activity of PP2A-Aα/B55α/Cα complex. Further, FAM122A potentiates the degradation of catalytic subunit PP2A-Cα with the increased poly-ubiquitination. In agreement, FAM122A silencing inhibits while its overexpression enhances cell growth and colony-forming ability. Collectively, we identify FAM122A as a new endogenous PP2A inhibitor and its physiological and pathophysiological significances warrant to be further investigated.

## INTRODUCTION

Complex processes in cell signaling require a set of molecular tools to modulate the activity and localization of specific proteins. Many of these responsibilities are regulated by protein phosphorylation. As the major regulatory mechanism employed by eukaryotic cells at the protein level, protein phosphorylation is dynamically controlled by kinases and phosphatases, whose opposite actions reversibly phosphorylate proteins [1]. Type 2A protein phosphatases (PP2A), which constitutes ~1% of total cellular protein, belongs to the phosphoprotein phosphatase (PPP) enzymes, which also comprise the serine(Ser)/threonine (Thr) phosphatases PP1, PP2B (calcineurin), PP4, PP5, PP6, and PP7 [2]. Together with PP1, the ubiquitously expressed PP2A phosphatases constitute the bulk of Ser/Thr phosphatase activity in a given tissue [2]. It dephosphorylates a plethora of cellular proteins involved in signal transduction besides regulating metabolic enzymes, including cell surface receptor molecules, cytosolic protein kinases, and transcription factors. The modification of these proteins important for different facets of cellular functions such as cell-cycle regulation, cell transformation, and cell fate determination affects essential processes of every cell [3–10]. Accordingly, dysregulations including overexpression or inactivation of PP2A result in developmental abnormality, embryonic lethality and neonatal death [11–13]. Also, mutation of PP2A subunits or decreased phosphatase activity of PP2A has been found in a variety of diseases including Alzheimer's disease, solid tumors and hematological malignancies [14–18], and the enhanced PP2A activity has also been found to be correlated with type II diabetes, inflammatory lung diseases like asthma and chronic obstructive pulmonary disease, and heart failure [19, 20]. Thus, PP2A can be as a favorable drugable target in the treatment of various cancers and relevant diseases [7].

Structurally, PP2A phosphatases are holoenzymes, constituting of at least two subunits [21, 22]. The PP2A core enzyme consists of a scaffolding subunit PP2A-A and a catalytic subunit PP2A-C. The A and C subunits each exist with an α and a β isoform in mammalian cells, which are encoded by two different genes each, giving rise to two highly related Aα and Aβ isoforms that share almost 87% sequence identity, and two nearly identical Cα and Cβ isoforms with 97% identity [23]. The PP2A-A subunit is composed of 15 tandem HEAT (named for the set of four cytoplasmic proteins Huntingtin, EF3, PP2A-A subunit and TOR1 which were first recognized to contain them) repeats and is expressed in excess of the other subunits in mammalian cells [24]. The C subunit binds to HEAT repeats 11–15 of the A subunit to form the PP2A core enzyme [25]. The core PP2A dimer can associate differently with each class of regulatory B-type subunits to form different PP2A heterotrimers. In mammals, there are four classes of B subunits including B (B55/PR55), B’ (B56/PR61), B’’ (PR48/PR72/PR130) and B’’’(PR93/PR110)/striatin, which are encoded by 15 different genes that result in 23 different isoforms through alternative gene promoters, alternative splicing or alternative translation [22]. Therefore, it is predicted that the PP2A holoenzyme has over 80 distinct combinations. Although the stoichiometry of PP2A within cells is poorly understood [26], there is evidence that approximately one-third of PP2A occurs as PP2A dimer, while the majority of PP2A-complexes are heterotrimers [21].

Like most protein phosphatases, PP2A are not all passive regulators for cell signaling. As reviewed [1, 22, 26], specific extracellular stimuli or stresses can either directly, or indirectly through activation of protein kinases, regulate PP2A activity by the generation of second messengers, such as cAMP, Ca^2+^ and ceramides. Several protein kinases such as the double stranded RNA-dependent protein kinase (PKR), extracellular signal-regulated kinase (ERK), DNA damage triggers ATM, DNA damage-induced Chk1 and protein kinase C (PKC) affect PP2A activity through phosphorylation of some B-type subunits. On the other hand, PP2A catalytic activity can be directly inhibited by an emerging set of specific cellular PP2A inhibitory proteins, including ANP32A (also called PP2A Inhibitor 1) [27], SET (also called PP2A Inhibitor 2) [17, 28], CIP2A (Cancerous Inhibitor of PP2A) [29] and cAMP-regulated phosphoproteins, ARPP16 and ARPP19, and the closely related α-endosulfine [30, 31] and TIP(type 2A interacting protein) [32]. Herein, we identify that FAM122A (family with sequence similarity 122A) or C9orf42 (chromosome 9 open reading frame 42) is a new endogenous PP2A inhibitor.

## RESULTS

### Physical interaction of FAM122A with PP2A-Aα/B55α/Cα complex

**Figure 1:**
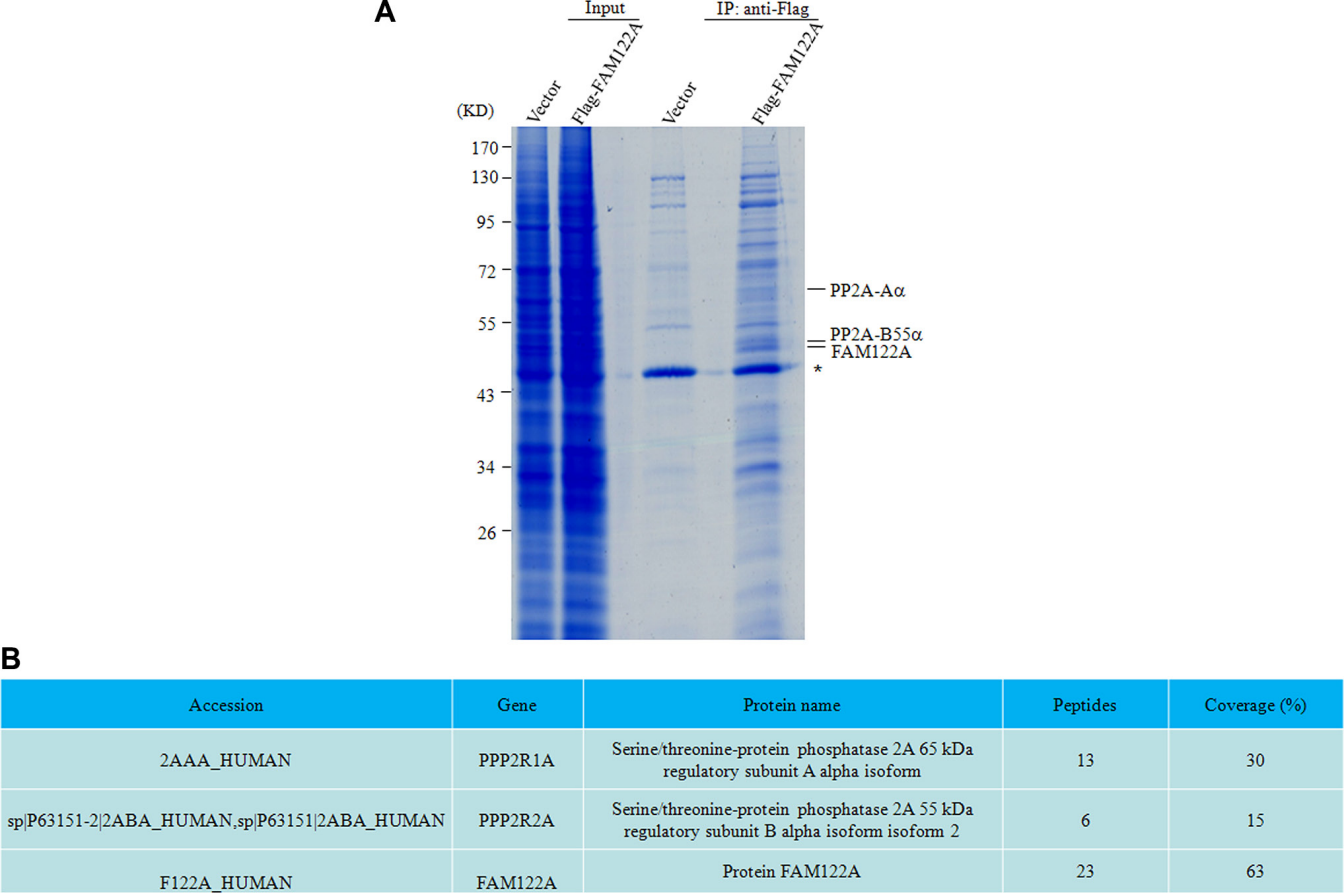
PP2A-Aα and B55α subunits were identified as the putative interactors with FAM122A by IP-MS (**A**) The gel graph depicting the input and IP products by anti-Flag M2 affinity gel in Flag-tagged FAM122A and Flag expressing 293T cells followed by staining with Coomassie Brilliant Blue. The symbol * indicates a non-specific band. (**B**) The proteins indicated as in A were analyzed by scaffold 4 proteome software and their information was presented. Peptides and coverage indicate the numbers of unique peptides and the percentage of sequence coverage in the identified proteins, respectively.

The *FAM122A* gene is localized on chromosome 9q21.1 within the intron 1 of *PIP5K1B* (phosphatidylinositol 4-phosphate 5-kinase) gene, and has 287 amino acids with highly sequence identity in a series of mammary animals including *bostaurus, canis lupus familiaris, macacamulatta, rattusnorvegicus* and *musmusculus*. However, the function of FAM122A is not clear, although its single nucleotide polymorphisms were found in chronic kidney disease [33, 34]. Also, genome-wide linkage scan showed that chromosomal region 9q21.1 had a suggestive linkage to the metabolic syndrome including cardiovascular disease and type 2 diabetes [35]. Therefore, we attempted to identify the potential FAM122A-interacting proteins. For this purpose, 293T cells were stably transfected with the empty or Flag-tagged FAM122A expressing plasmids, and the cell lysates were immunoprecipitated (IP) with anti-Flag M2 affinity gel, followed by the competitive elution of 3× Flag peptide. The eluted immunoprecipitates were run on SDS-PAGE and stained by Coomassie Brilliant Blue (Figure 1A). Differential protein bands between Flag-tagged FAM122A and empty vector-expressing cells were individually excised, digested and analyzed by HPLC/mass spectrometry. Of great significance, PP2A-Aα and PP2A-B55α proteins (Figure 1B) were found in immunoprecipitates from Flag-tagged FAM122A but not empty vector-expressing cells, although there also seems to be increased proteins in the input for the Flag-FAM122A (Figure 1A). An early report showed that FAM122A was also in a total of 197 protein interactions with the human PP2A system, which were identified by an integrated systems biology method [36]. On the other hand, PP2A participates in a variety of biological processes including cell growth, proliferation, differentiation and apoptosis [37]. Therefore, in this work we focused on the potential interaction of FAM122A with PP2A complex and their functional significance.

**Figure 2:**
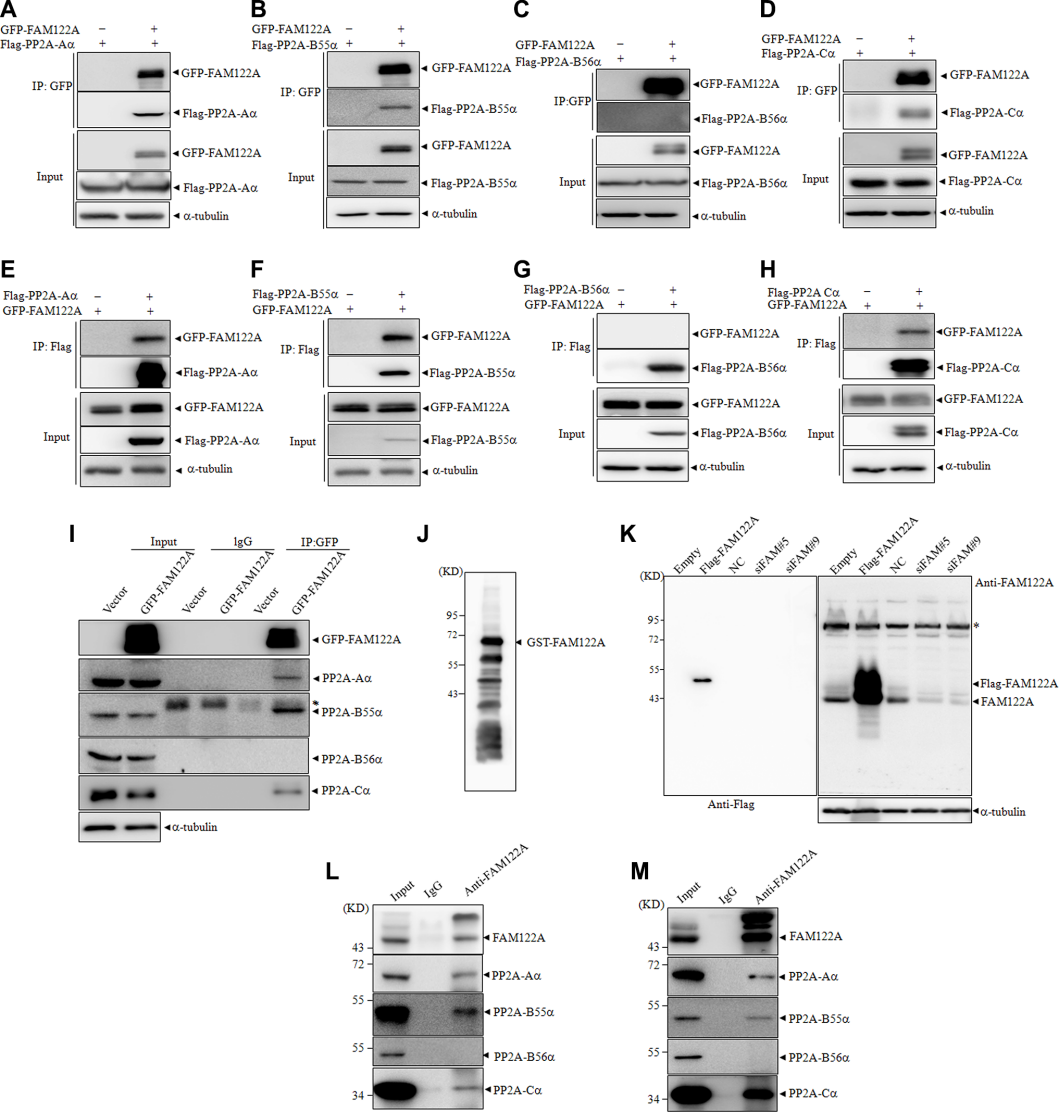
FAM122A interacts with PP2A complex (**A**–**H**) 293T cells were transiently cotransfected with indicated plasmids followed by IP with anti-GFP antibody (A–D) or anti-Flag antibody (E–H). The indicated proteins were examined by western blot. (**I**) 293T cells were transiently transfected with GFP-tagged FAM122A or GFP expressing plasmids and IP was performed by anti-GFP antibody or non-specific IgG. Subunits of PP2A complex were detected by western blot. The symbol * indicates heavy chains of immunoglobulin. (**J**) The recombinant GST-FAM122A was blotted with the anti-FAM122A antibody. (**K**) 293T cells were stably transfected with Flag-FAM122A and empty plasmids, or with two independent shRNAs targeting FAM122A and non-specific shRNA as a negative control (NC). Western blot was applied to examine the specificity of anti-FAM122A antibody. The symbol * indicates nonspecific bands. (**L**, **M**) 293T (L) and Jurkat (M) cells were immunoprecipitated with anti-FAM122A antibody or non-specific IgG followed by western blot with indicated antibodies. 10% cell lysates (input) was used as a positive control.

To confirm the interaction of FAM122A with PP2A subunits, 293T cells were transiently co-transfected with GFP-tagged FAM122A or empty vector and Flag-tagged PP2A subunit Aa, B55α, B56α or Cα. Forty-eight hours post-transfection, co-IP with anti-GFP antibody was performed. The results showed that the anti-GFP antibody, which efficiently precipitated GFP-tagged FAM122A protein, could effectively pulled down Flag-tagged PP2A-Aα, B55α and Cα, but not B56α (Figure 2A–2D). Reciprocally, we also transiently co-transfected these four Flag-tagged PP2A subunits or empty vector together with GFP-tagged FAM122A into 293T cells, followed by co-IP assay with anti-Flag M2 affinity gel. In agreement, GFP-tagged FAM122A could also be significantly co-precipitated by Flag-tagged PP2A-Aα, B55α and Cα but not B56α (Figure 2E–2H). Next, we detected whether GFP-tagged FAM122A interacts with endogenous PP2A subunits in GFP-tagged FAM122A or empty vector-transfected 293T cells. As depicted in Figure 2I, anti-GFP antibody could pull down endogenous PP2A-Aα, B55α and Cα but not B56α proteins together with GFP-tagged FAM122A.

**Figure 3:**
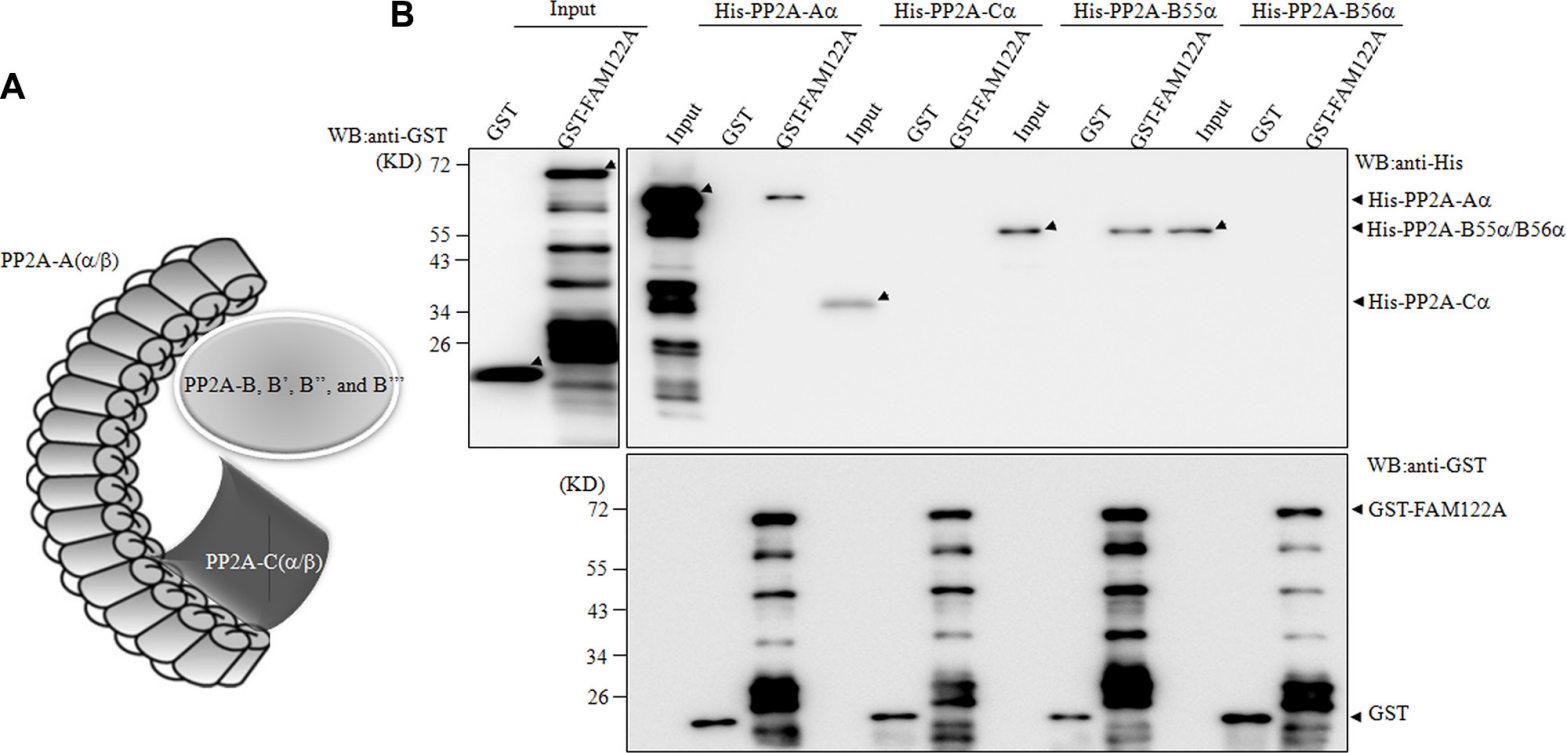
FAM122A interacts directly with PP2A-Aα and B55α subunits (**A**) The graph indicats PP2A heterotrimer. (**B**) *E. coli BL21* expressing GST and GST-FAM122A proteins were purified and incubated with His-tagged PP2A subunits. The GST pull down complexes were blotted with anti-His (top right panel) or anti-GST antibody (bottom).

To further test whether endogenously expressed FAM122A interacts with these PP2A subunits, we prepared anti-FAM122A antibody by using *Escherichia coli* BL21 expressed GST-tagged FAM122A protein to immunize New Zealand white rabbit. The antibody was affinity purified by ImmunoPure IgG Purification Kit. As shown in Figure 2J, the antibody effectively identified the recombinant FAM122A protein, in spite of multiple GST-tagged FAM122A proteins with differents length, which could also be seen in anti-GST-based blot (see Figure 3B), possibly due to incomplete translation in E. coli. The specificity of the anti-FAM122A antibody was also confirmed in Flag-tagged FAM122A expressing and silencing 293T cells (Figure 2K). Furthermore, the anti-FAM122A antibody-based IP assay also confirmed the interaction of endogenous FAM122A and PP2A-Aα, B55α, and Cα but not B56α proteins in 293T (Figure 2L) and Jurkat cells (Figure 2M), a human acute T cell leukemia cell line.

### Direct binding of FAM122A with PP2A-Aα and B55α but not Cα

Because PP2A-A, B and C subunits forms a complex (Figure 3A), we attempted to determine which PP2A subunit(s) FAM122A interacts with. To this end, the recombinant glutathione S-transferase (GST) alone or GST-tagged FAM122A was incubated with the *in vitro* translated His-tagged PP2A subunits in *E. coli*, followed by GST pull down assay. In the GST pulled-down complex or input, anti-GST antibody-based immunoblot showed multiple GST-tagged FAM122A proteins with different length (Figure 3B), like that seen in anti-FAM122A antibody-based blot (Figure 2J). Regardless of this, the recombinant GST-tagged FAM122A rather than GST alone could effectively pull down His-tagged PP2A-Aα and B55α but not Ca and B56α (Figure 3B), suggesting that FAM122A interacts directly with PP2A-Aα and B55α subunits.

### FAM122A is an inhibitor of PP2A-Aα/B55α/Cα heterotrimer

**Figure 4:**
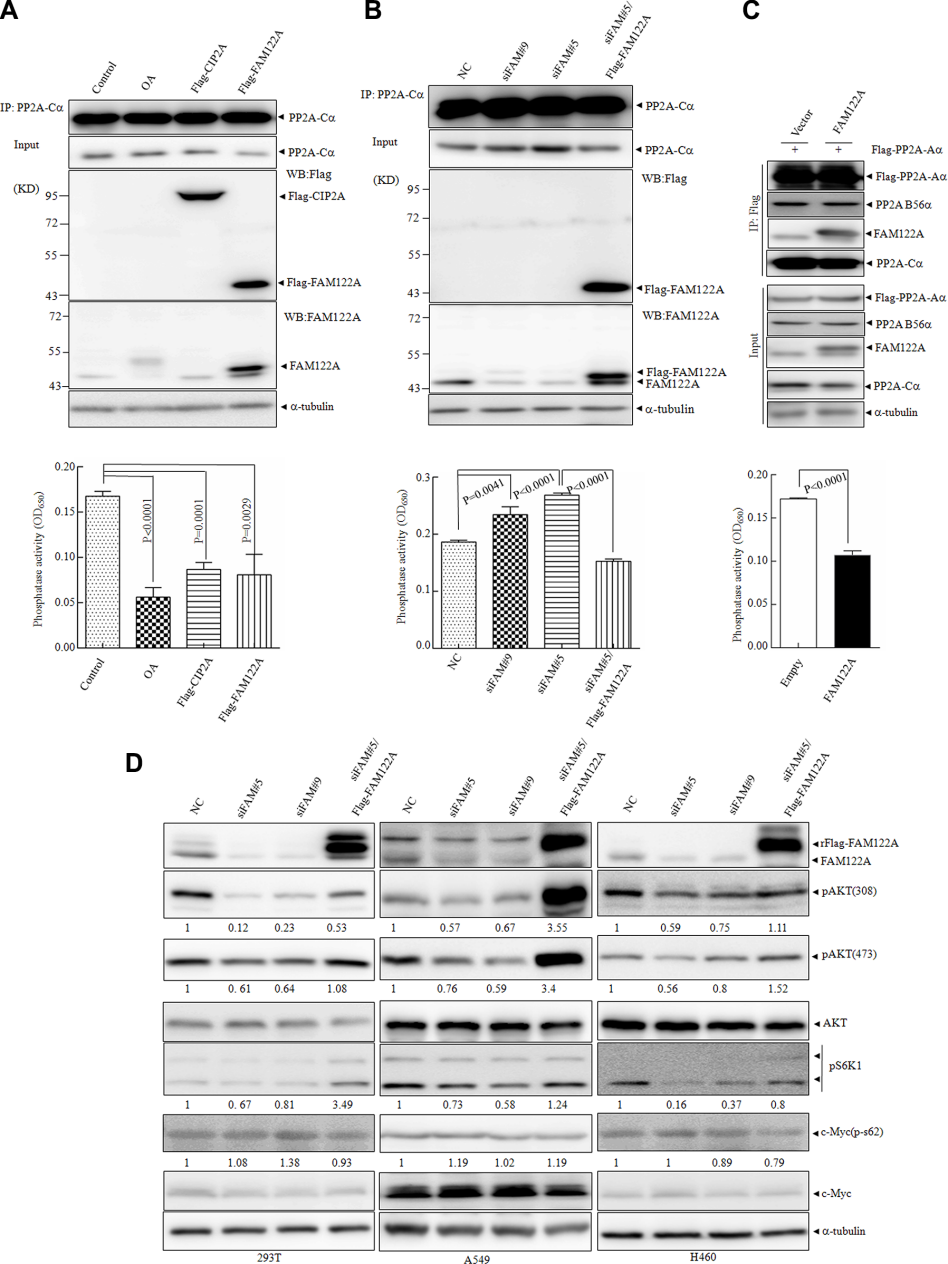
Effects of FAM122A on PP2A phosphatase activity (**A**, **B**) The cellular lysates of 293T cells transiently transfected with Flag tagged FAM122A or CIP2A, or treated with OA at 50 nM for 6 hours (A) and FAM122A silencing 293T cells, together with NC and siFAM#5/Flag-FAM122A cells (B) were respectively immunoprecipitated with anti-PP2A-Cα antibody (top panel) followed by *in vitro* phosphatase activity assay (bottom panel). (**C**) Stably expressing FAM122A or empty 293T cells were transiently transfected with Flag-PP2A-Aα, followed by IP with Flag antibody (upper panel) and phosphatase assay (bottom panel). (**D**) FAM122A silencing and Flag-FAM122A-reexpressing cells as indicated were examined for phosphorylated state of several PP2A substrates with a-tubulin as a loading control. The relative ratios to control cells of phosphorylated proteins against a-tubulin were shown.

The interaction of FAM122A with PP2A complex promoted us to detect whether FAM122A modulates the phosphatase activity of PP2A. For this purpose, an *in vitro* phosphatase assay was applied to the anti-PP2A-Cα antibody-pulled down precipitates from equal amounts of 293T cells respectively with ectopic expression of Flag-tagged FAM122A and CIP2A (a known PP2A inhibitor) or with the treatment of 50 nM of okadaic acid (OA, a chemical PP2A inhibitor) [38]. The anti-PP2A-Cα antibody precipitated PP2A-Cα protein with similar effects in these treated or transfected cells (top panels, Figure 4A), although FAM122A overexpression reduced PP2A-Cα protein in whole lysates (input panels, Figure 4A). Like OA treatment and CIP2A overexpression, FAM122A overexpression also significantly decreased PP2A activity (bottom panel, Figure 4A). Of note, OA appeared to induce modification of FAM122A, while CIP2A had no impact on FAM122A expression (top panel, Figure 4A). The same assay was also used in 293T cells which were transfected by two pairs of siRNAs specifically targeting FAM122A (designated siFAM#9 and siFAM#5) together with a non-specific siRNA (NC). Like that seen in ectopic expression of FAM122A (Figure 4A), FAM122A silencing increased PP2A-Cα protein in whole lysates (input panels, Figure 4B), but similar amounts of PP2A-Cα proteins were precipitated in these treated or transfected cells (top panel, Figure 4B). And FAM122A silencing (top panels, Figure 4B) remarkably enhanced phosphatase activity of PP2A (bottom panel, Figure 4B), which could be rescued by re-expression of Flag-tagged and siFAM#5-resistant FAM122A (bottom panel, Figure 4B). Notably, FAM122A transfection overwhelmingly enhanced its expression level far above the basal level, but only restored the phosphatase activity to the basal level in siRNA treated cells (bottom panel, Figure 4B). We also applied the *in vitro* phosphatase assay to the anti-Flag antibody-pulled down precipitates from equal amounts of 293T cells with ectopic expression of Flag-tagged PP2A-Aα in the presence and absence of FAM122A overexpression. The results showed that FAM122A overexpression failed to impact the PP2A-Aα/B56α interaction, and it still inhibited PP2A activity in the PP2A-Aα pulled down precipitates (Figure 4C).

It has been well known that the specific substrates of PP2A are dependent on its different B subunits [23]. The fact that FAM122A interacts with PP2A-B55α but not B56α suggests that FAM122A may modulate the phosphorylation state of PP2A substrates specifically targeted by PP2A-B55 subunit. Akt has been identified as an important PP2A-B55-specific substrate [39], and S6K1 as a downstream substrate of AKT/mTOR signaling regulated by PP2A [40, 41], while c-myc is specifically targeted by PP2A-B56 [29, 42]. For this purpose, three cell lines with detectable FAM122A protein, including human embryonic kidney 293T cells, human lung carcinoma epithelial cells A549 and large cell lung cancer epithelial cells H460, were stably transfected by these two specific siRNAs. The effective knock down effect of FAM122A has been confirmed by western blot in three cell lines (Figure 4D) and 293T cells (Figure 2K). Then, we detected the phosphorylated states of AKT, S6K1 and c-myc in 293T, A549 and H460 cells with or without FAM122A silencing. The results revealed that FAM122A silencing could significantly inhibit the phosphorylated AKT at Thr308 and Ser473 and S6K1 but not c-myc in all three cell lines, and the inhibited AKT and S6K1 phosphorylations could be restored by re-expression of FAM122A (Figure 4D). Collectively, all these data support that FAM122A is a specific inhibitor of PP2A-Aα/B55α/Cα heterotrimer.

### FAM122A enhances degradation of PP2A-Cα protein by the ubiquitination

**Figure 5:**
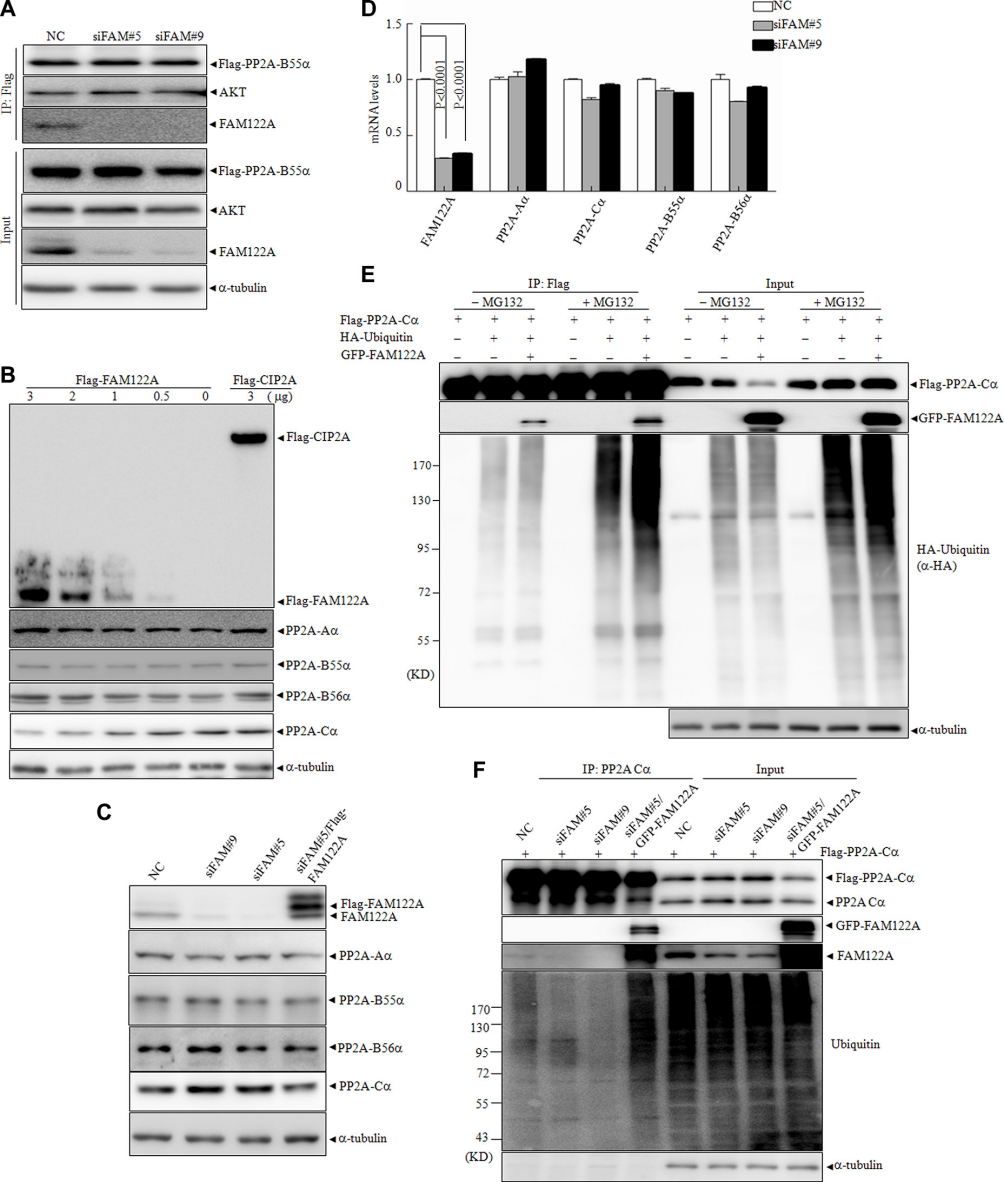
Effects of FAM122A on PP2A subunit expression and the ubiquitination of PP2A-Cα protein (**A**) FAM122A silencing and NC 293T cells were transiently transfected with Flag-PP2A B55α plasmid, followed by IP by anti-Flag M2 affinity gel. (**B**) 293T cells were transiently transfected with different concentrations of Flag tagged FAM122A or 3 μg Flag-CIP2A expressing plasmids. After transfection for 48 hours, the cell lysates were extracted and examined by western blot. (**C**) The protein levels of PP2A subunits from indicated 293T cells were detected by western blot. (**D**) The mRNA levels of PP2A subunits were examined by real-time PCR in the indicated 293T cells. (**E**) 293T cells were transiently cotransfected with the plasmids as indicated. After transfection for 48 hours, the cells were treated with or without MG132 at 20 μM for 4 hours. The cellular lysates were collected and immunoprecipitated with anti-Flag M2 affinity gel followed by western blot. (**F**) The indicated FAM122A silencing 293T cells together with NC or ectopically expressed GFP-FAM122A in siFAM#5 expressing cells were transiently transfected with Flag-PP2A-Cα plasmid respectively followed by the treatment of MG132 at 20 mM for 4 hours. IP was applied by anti-PP2A-Cα antibody and the endogenous ubiquitination of PP2A-Cα protein was analyzed by western blot.

The previous investigations showed that PP2A activity can be regulated by the alteration of protein expression of its subunits, post-translational modifications of PP2A-C subunit including phosphorylation and methylation, and the association of B subunits with the corresponding substrates [1, 23]. To investigate how FAM122A modulates the phosphatase activity of PP2A, we asked whether FAM122A expression affects the interaction of PP2A-B55 with its substrate Akt. For this purpose, Flag-tagged PP2A-B55α was ectopically expressed in siFAM#5, siFAM#9 or NC-infected 293T cells, followed by co-IP with anti-Flag M2 affinity gel. The results showed that the silence of FAM122A failed to alter PP2A-B55α/Akt interaction (Figure 5A). On the other hand, FAM122A overexpression neither changed PP2A-Aα and PP2A-B55α protein level nor induced the shift in the gel of PP2A-Cα (input, Figure 2I). However, our further investigation showed that Flag-tagged FAM122A dose-dependently decreased the PP2A-Cα with no changes for Aa and B55α or B56α subunits, which was not found in CIP2A overexpressing cells (Figure 5B). In agreement, FAM122A silencing did significantly increase PP2A-Cα protein, which could be antagonized by re-expression of FAM122A (Figure 5C). We also demonstrated that FAM122A silence did not affect the PP2A-Cα mRNA expression (Figure 5D). Moreover, GFP-tagged FAM122A overexpression also reduced ectopically expressed Flag-tagged PP2A-C protein level to a degree (Figure 2D).

The above results suggest that FAM122A promotes the degradation of PP2A-Cα protein and/or causes post-translational modifications of PP2A-Cα protein which alters its sensitivity to monoclonal anti-PP2A-Cα antibody. Accumulating lines of evidence show that PP2A-C can be regulated by the ubiquitin system [43]. To test whether PP2A-Cα ubiquitination was affected by FAM122A, Flag-tagged PP2A-Cα, HA-ubiquitin and/or GFP-tagged FAM122A were transiently transfected into 293T cells in the presence or absence of proteasome inhibitor MG132. The results demonstrated that FAM122A overexpression significantly reduced Flag-tagged PP2A-Cα protein, which could be effectively rescued by MG132 treatment (input channel, Figure 5E), suggesting that FAM122A induced PP2A-Cα degradation by proteasome. Further, co-IP assay showed that FAM122A remarkably increased the poly-ubiquitin- PP2A-Cα protein in the presence of MG132 (IP channel, Figure 5E), as determined by anti-HA antibody. These data propose that FAM122A induces degradation of PP2A-Cα protein by the poly-ubiquitin/proteasome system, which could also be confirmed in FAM122A-silencing cells (Figure 5F).

### Suppression of cell growth and colony formation in FAM122A silencing cells

**Figure 6:**
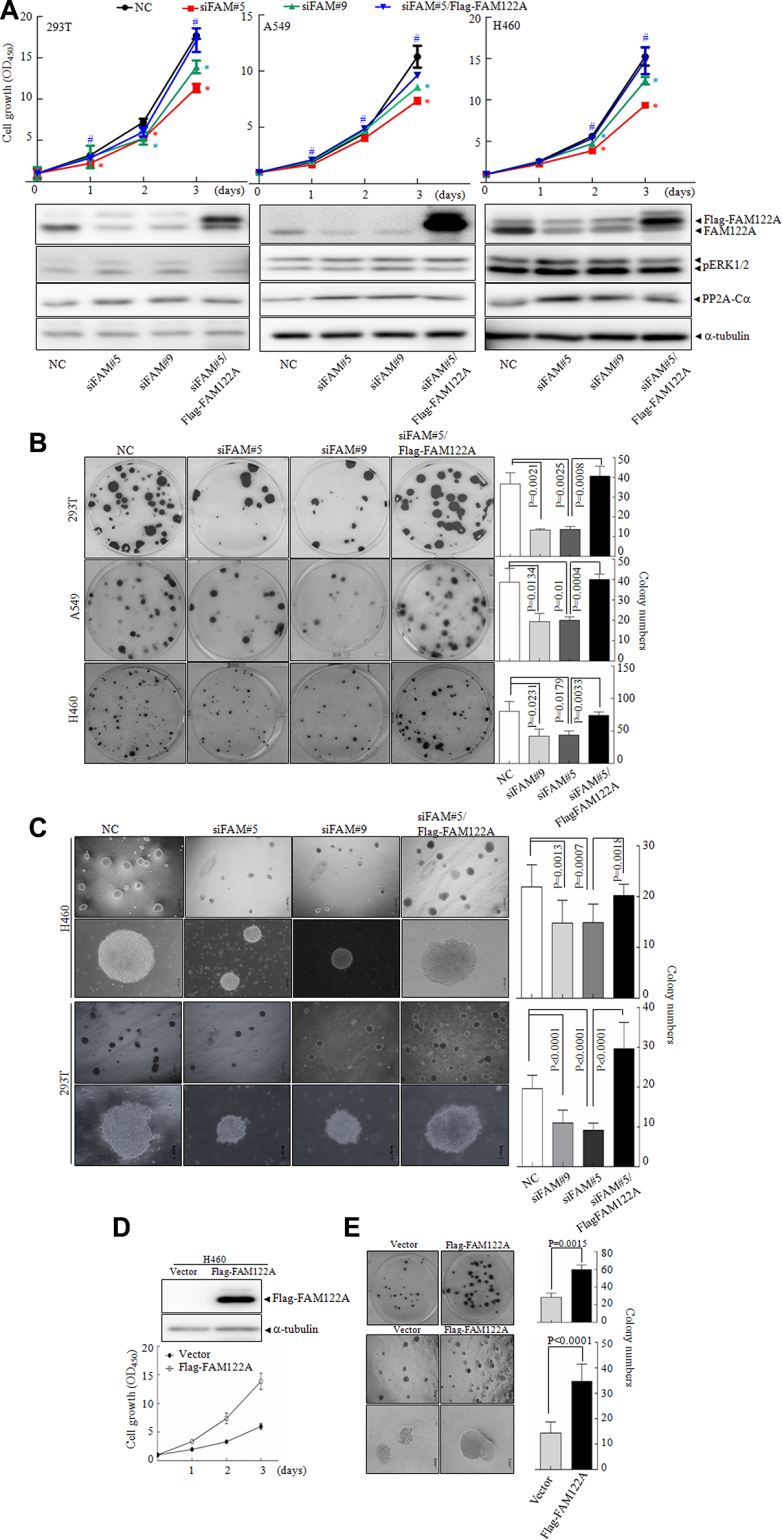
The effects of FAM122A on cell growth and colony formation (**A**) Three indicated FAM122A silencing cells together with the stable expression of Flag-FAM122A in siFAM#5 expressing cells were respectively cultured and cell growth was monitored. Data indicate means with bar as S.D of triplicate samples in an independent experiments. The symbol * indicated *p* < 0.05 compared with NC cells and the symbol # implied *p* < 0.05 compared with that of siFAM#5 expressing cells. The indicated proteins were tested with the corresponding antibodies. (**B**, **C**) The cells were cultured in plates (B) and in soft agar (C) for two weeks to observe the colony formation. (**D**–**E**) The cell growth was examined in ectopically expressing Flag-FAM122A and empty H460 cells by CCK-8 (D), plate colony (the upper panel of E) and soft agar colony formation (the bottom panels of E). The graphs on right panel were statistically analyzed. The scale bar represents 500 μm (top row) or 100 μm (bottom row) in H460 and 293T cells (C and bottom panels of E).

Although it was reported that different PP2A trimers can negatively regulate activity of all mitogen-activated protein kinase (MAPK) such as ERKs, here we showed that FAM122A silencing did not significantly regulate the phosphorylation of ERK1/2 (Figure 6A), which is consistent to the notion that PP2A-B56 but not PP2A-B55 increases ERK dephosphorylation [44]. As well known, PP2A dephosphorylates over 300 substrates involved in the cell cycle and regulates almost all major pathways and cell cycle checkpoints, thus playing a critical multi-faceted role in the regulation of the cell proliferation [45]. Therefore, we addressed whether FAM122A could affect cell growth. Thus, the cell growth of FAM122A silencing cells was monitored by CCK-8 assay. The results showed that FAM122A knockdown remarkably inhibited the growth of all these three cell lines, particularly on the culturing days 2 and 3 (top panels, Figure 6A). Moreover, FAM122A silencing also abrogated the cell colony formation (Figure 6B) and significantly decreased the numbers and sizes of colony growth in soft agar (Figure 6C). To confirm that these effects are FAM122A silencing-specific, we re-expressed Flag-tagged and siFAM#5-resistant FAM122A into these three cell lines and the results demonstrated that FAM122A re-expression could rescue the reduced growth and colony formation in siFAM#5 expressing cells (Figure 6A–6C). Additionally, ectopic expression of FAM122A promoted the growth and colony formation in H460 cells (Figure 6D–6E). Cumulatively, our results suggest that FAM122A promotes cell growth, which is consistent with the effect of suppressed PP2A activity.

## DISCUSSION

PP2A protein complexes are involved in many cellular processes, including cell growth, differentiation, apoptosis, cell motility, DNA damage response and cell cycle progression [7, 46]. In this study, with co-IP-based proteomic strategy, we demonstrated that FAM122A protein interacted with PP2A complex, which could be confirmed in cells with over-expressions of FAM122A and/or PP2A subunits. Especially, endogenous FAM122A and PP2A could also interact with each other, proposing their physiological interaction. Considering that the PP2A-Aα isoform subunit constitutes about 90% of PP2A holoenzymes while Aβ isoform forms 10% of PP2A enzyme [47], and the Cα isoform is also expressed in higher abundance than βisoform [23], here we mainly focused on their α subunits. Our GST pulldown assay showed that FAM122A directly interact with PP2A-Aα and PP2A-B55α rather than B56α and PP2A-Cα. Based on these findings, we demonstrated that FAM122A protein could effectively inhibit phosphatase activity of PP2A-Aα/Cα/B55α but not PP2A-Aα/Cα/B56α complex. It has well been known that within PP2A complex, the B subunits, usually expressing in a cell- or tissue-specific way with distinct subcellular localizations, determine the substrate specificity of PP2A holoenzyme, thus directly modulating PP2A activity [1, 22]. Our results showed that FAM122A enhanced the phosphorylation of B55-specific substrates Akt and not B56-specific substrates c-myc and ERK1/2, further supporting the specific inhibiting activity of FAM122A on B55-forming PP2A activity. Further, three human cell lines with FAM122A silencing presented significantly reduced growth and colony formation, which is consistent with the notion that the enhanced PP2A activity can suppress cell growth [45].

To understand how FAM122A protein inhibits phosphatase activity of PP2A-Aα/Cα/B55α complex, we showed that FAM122A dose-dependently decreased the PP2A-Cα, and FAM122A silencing did significantly increase PP2A-Cα protein. These results suggest that FAM122A promotes the degradation of PP2A-Cα protein and/or causes post-translational modifications (PTMs) of PP2A-Cα protein, the latter leading to the alteration of its binding capacity with anti-PP2A-C monoclonal antibody, since this antibody is specifically against the C-terminal amino acid residues 295–309 that are the main PTM sites of PP2A-C subunit. Here we demonstrated that the proteasome inhibitor MG132 effectively antagonized FAM122A overexpression-induced decrease of PP2A-Cα protein, suggesting that FAM122A can degrades PP2A-Cα protein by proteasome system, although its exact mechanisms remain to be explored. Regardless of this, our *in vitro* phosphatase assay demonstrated that FAM122A effectively inhibited the PP2A activity in the PP2A-Cα-pulled down precipitates with the same amounts of the PP2A-Cα complex, suggesting that the inhibitory activity of FAM122A involves its interaction with PP2A complex besides decreased PP2A-Cα. Additionally, it is also possible that FAM122A competes with PP2A-Cα to bind with PP2A-Aα complex to regulate PP2A activity, because FAM122A interacts with PP2A-Aα and B55α rather than PP2A-Cα subunits.

It is well known that the monomeric C subunit is unstable and requires binding to the A subunit or other non-canonical B subunits to preserve its activity [48]. Amounting evidence shows that PP2A phosphatase activation is regulated by PTMs of PP2A-C subunit protein, B subunit diversity, the association of regulatory subunits with substrate proteins or binding with α4 [49, 50], an important regulator of PP2A activity. The current investigations have shown that the C-terminus of PP2A-Cα undergoes methylation at L309 by the regulation of two enzymes, methyltransferase LCMT-1 that is expressed in the cytoplasm and a lipase-like methylesterase PME-1 that is expressed in the nucleus [1]. On the other hand, the phosphorylation of multiple residues on the C terminus of PP2A-C is also critical for modulating PP2A activity via B subunit interactions. A recent report showed that ubiquitin E3 ligase NOSIP induces the ubiquitination of PP2A-C subunit and the loss of *NOSIP* enhances PP2A activity, resulting in perinatal lethality [51]. Also, progestin-inducible EDD E3 ubiquitin ligase could polyubiquitinate PP2A-C by binding to the C-terminal of α4 [52]. Here we also demonstrated that FAM122A potentiates poly-ubiquitination of anti-PP2A-Cα precipitates, which suggests that FAM122A enhances ubiquitination of PP2A-Cα and/or its partner(s) such as CIP2A, SET and TIPRL. To address this, *in vitro* ubiquitination assay by using recombinant PP2A-C protein deserves to be performed. Also, it remains to be further investigations how FAM122A induces the polyubiquitination of PP2A-Cα or its partners. On one hand, amino acid sequence analysis revealed that FAM122A is ~31% homologous to NEDD4 (data not shown), a founding member of a family of ubiquitin-protein ligases [53]. Therefore, FAM122A itself has ubiquitin-E3 ligase activity deserved to be explored, although FAM122A has no direct interaction with PP2A-Cα. On the other hand, several PP2A-C-targeting ubiquitin-E3 ligases such as Mid1 and EDD [52–54] have been reported. Whether FAM122A indirectly impacts activities of these specific E3 ligase and/or their regulators such as α4 and PME-1 [43, 54, 55] to induce polyubiquitination also needs to be further investigated.

In summary, our study shows that FAM122A directly interacts with Aα and B55α subunits of PP2A and inhibits its phosphatase activity, and its biological and pathological significances warrant to be further explored.

## MATERIALS AND METHODS

### Cell culture and transfections

Human cervical carcinoma cell line HeLa, human lung cancer cell lines A549 and H460 as well as 293T cells were purchased from the American Type Culture Collection (ATCC^™^). HeLa, 293T and A549 cells were cultured in Dulbecco's modified Eagle's medium (DMEM, HyClone, SH30022.01B) containing 1% penicillin and 1% streptomycin, supplemented with 10% FBS. H460 and Jurkat cells were cultured in RPMI 1640 medium (Sigma-Aldrich, St Louis, MO) supplemented with 10% fetal bovine serum (FBS, Gibco, 26140). All cells were incubated in 5% CO2/95% air humidified atmosphere at 37°C. For transient transfection, Lipofectamine 2000 transfection reagent (Invitrogen, Carlsbad, CA) was used following manufacturer's protocol. For cell transduction, retroviruses or lentiviruses were prepared through transient cotransfection with the expressing and helper plasmids into 293T cells by HilyMax Transfection Reagent according to the manufacturer's procedures (Dojindo Molecular Technologies, H357).

### Plasmids and shRNAs

Flag-FAM122A and GFP-FAM122A were constructed by inserting FAM122A cDNA into the PLVX-IRES-Puro and pEGFP-C1 vectors, respectively. Plasmids expressing Flag tagged PP2A-Aα, B55α, Ca, B56α and CIP2A were generated by inserting the cDNAs of PP2A-Aα, B55α, Ca, B56α and CIP2A into pCMV-3Flag vector by PCR strategy from the original plasmids including PP2A-Aα (pCool), B55α (pCool) and Cα (pVL1393) or constructed by ourselves. These original plasmids were kindly provided by Dr. Yi-Gong Shi from Tsinghua University. For GST-tagged FAM122A plasmid, FAM122A cDNAs was cloned and inserted into pGEX-5X-1 vector. Plasmids expressing His tagged PP2A-Aα, B55α, Ca and B56α were generated in pET-28a(+) vector with similar strategy as Flag tagged PP2A-Cα constructs. HA-tagged ubiquitin plasmid was generated by inserting ubiquitin cDNAs into pCDNA3.0 vector. These generated plasmids were confirmed by sequencing. SiRNA-resistant FAM122A plasmids with Flag or GFP tag were generated by QuickChangeTM Lightning Site- Directed Mutagenesis Kit. Pairs of complementary oligonucleotides against FAM122A were synthesized by Invitrogen (Shanghai), annealed and ligated into pSIREN-RetroQ Vector (Clontech Laboratories). These siRNA-carrying retroviruses produced in 293T cells. The target sequences for FAM122A were shown, respectively, as following: 5′-CACCAGATCAAACAAGAA-3′ for siFAM122A#5, 5′-CATTAGACCAAGTGTTCT -3′ for siFAM122A#9.

### Pull-down and MS analysis of Flag-FAM122A-bound proteins

About 3 × 10^7^ cells for transfection with indicated plasmids, 293T cell lysates were harvested. After brief sonication, the cell lysates were centrifuged with 12000 rpm at 4°C for 10 minutes. The lysates were pre-cleaned with normal IgG at 4°C for 2 hours and supernatants were incubated with anti-Flag M2 Affinity Gel (Sigma, A2220) overnight at 4°C. After immunoprecipitation, the beads were washed with lysis buffer without inhibitors for five times followed by three 1 ml Tris Buffered Saline (TBS) washes. Proteins were eluted with two 50 μl 150 μg/ml 3 × Flag-peptide (Sigma) in TBS for 30 minutes. Proteins in each elution were precipitated with cold acetone and the resulting pellet was washed 2 times with cold acetone, and the elutions were pooled for a final volume of 100 μl, the eluted proteins were separated by SDS-PAGE and visualized by coomassie brilliant blue (CBB) staining. The protein-containing band in the gel was excised, followed by in-gel digestion and analysis by LC-MS/MS as described previously [56]. The results were further analyzed by Scaffold 4 proteome software (Portland, OR), which integrates both Protein Prophet and Peptide Prophet. Two unique peptides must be identified independently for each protein, the peptide probability must be 95% or higher, and the protein probability must be 95% or higher.

### Immunoprecipitation

Cells were harvested and lysed with immunoprecipitation buffer (50 mM Tris–HCl, pH 7.6; 150 mM NaCl; 1 mM EDTA; 1% NP-40; 1% protease inhibitor cocktail; 1 mM PMSF). After brief sonication, the lysates were centrifuged at 12000 g for 10 minutes at 4°C. For immunoprecipitation of Flag-tagged proteins, the supernatants were incubated with anti-Flag M2 Affinity Gel (Sigma-Aldrich) at 4°C overnight. For immunoprecipitation of GFP-tagged proteins, supernatants were incubated with anti-GFP Affinity Gel (MBL) at 4°C overnight. Otherwise, supernatants were incubated with indicated antibodies overnight and protein A/G-agarose beads (Santa Cruz, CA) for 2 hours at 4°C. The precipitates were washed three times with immunoprecipitation buffer, boiled in sample buffer, and subjected to immunoblot assay.

### GST Pull-down assay

GST and GST-FAM122A proteins were expressed in *E. Coli BL21* by induction with Isopropyl β-D-1-thiogalactopyranoside (IPTG) at 22°C overnight and purified with GST Bind Resin (Novagen). HIS-tagged PP2A-Aα, B55α, Ca and B56α proteins were respectively expressed in *BL21* by induction with 0.2 mM IPTG at 22°C for 6 hours. The purified GST or GST-FAM122A was incubated with HIS tagged proteins. Then the precipitations were eluted by SDS sample buffer and analyzed by western blots.

### PP2A phosphatase activity assay

PP2A activity was measured by Ser/Thr phosphatase assay kits (Upstate Biotechnology) according to manufacturer's instructions. Cells were washed in TBS and lysed in phosphatase assay buffer (20 mM imidazole-HCl, 2 mM EDTA, 2 mM EGTA, 0.1% NP-40, pH 7.0, 1% protease inhibitors) on ice. PP2A-Cα protein was immunoprecipitated by 4 μg anti-PP2A-Cα antibody (clone 1D6, Upstate) and protein A/G agarose for 2 hours at 4°C in 500 μg total cell lysates. The immunoprecipitated protein was incubated with synthetic phosphopeptide K-R-pT-I-R-R for 10 minutes at 30°C followed by detecting PP2A activity with malachite green phosphate detection solution. For confirming IP efficiency, the amount of immunoprecipitated PP2A was also monitored by anti-PP2A-Cα antibody with western blot.

### Quantitative real-time PCR with reverse transcription

Total RNA was isolated by a Trizol kit (Invitrogen) with DNase (Promega) according to manufacturer's instructions. A total of 2 μg RNA was subjected to reverse transcription to synthesize cDNA using M-MLV reverse transcriptase (Promega, Fitchburg, WI). Quantitative real-time PCR for FAM122A, PP2A-Aα, -B55α, -B56α and –Cα together with β-actin was performed with SYBR Green PCR Master Mixture Reagents (Applied Biosystems) on the ABI PRISM 7900 system (Applied Biosystems). Paired primers were used as follows.

**Table:** Primers for quantitative real-time PCR

Genes	Forward primers	Reverse primers
FAM122A	AAGATGGAGCTAGACCTGGAG	CCGGCGAAGTGTCACTGAG
PP2A Aα	TAGACGAACTCCGCAATGAGG	CACCAGGGTAGTGAAGGTTCC
PP2A B55α	CATACCAGGTGCATGAATACCTC	GGGTTATGTCTCGCTTTGTGTTT
PP2A B56α	AGAGCCCTGATTTCCAGCCTA	TTTCCCATAAATTCGGTGCAGA
PP2A Cα	CAAAAGAATCCAACGTGCAAGAG	CGTTCACGGTAACGAACCTT
β-actin	CATCCTCACCCTGAAGTACCC	AGCCTGGATAGCAACGTACATG

### Western blots, antibodies and reagents

The whole cell lysates, extracted in PBS plus 2 × SDS, were equally loaded onto SDS-PAGE and subsequently transferred to PVDF transfer membranes (Millipore Corporation Billerica, MA). After blocking in 5% non-fat milk at room temperature for 1 hour, the membranes were incubated with indicated primary antibodies overnight at 4°C, followed by HRP-linked secondary antibodies (Cell Signaling, 7074). The signals were detected by chemiluminescence phototope-HRP kit (Millipore, WBKLS0500) according to the manufacturer's instructions. Anti-PP2A-Aα (#07-250), B55α (#05-592), B56α (#07-1221) and Cα (#05-421) antibodies used in western blot were purchased from Millipore. Antibodies against pAkt S473(#4060), pAkt T308(#2965), AKT(#9272), pS6K1 Thr389(#9234s), pERK1/2 Thr202/Tyr204(#9101) and HIS (#2366) were purchased from Cell Signaling Technology. The antibody for HA (26D11) was from Abmart. GST (ab111947) and c-Myc (p-s62) were from Abcam. Antibody against c-Myc was from Santa Cruz. Anti-a-tubulin-HRP-DirecT antibody (PM054-7) and anti-DDDDK-tag (mouse, M185-3L) were purchased from Medical and Biological Laboratories. Anti-FAM122A antibody was made from ABclonal Company. Okadaic acid (O7760-10UG) was purchased from Sigma-Aldrich.

### Colony formation assay and anchorage-independent growth

100 293T or H460 cells and 200 A549 cells, stably expressing indicated plasmids, were respectively seeded on 6-well plates. The cells were cultured *in vitro* for 14 days and stained with 1% crystal violet after fixation with methyl alcohol. All visible colonies were counted in the plates. For anchorage-independent growth assays, 5000 293T or H460 cells were respectively seeded on 6-well plates and cultured in 0.3% soft agar medium containing 10% FBS for 14 days. Visible colonies were counted following random observation in 5 fields. The number and size of colonies were analyzed by ImageJ software from microscopy images.

### CCK-8 assay

Plates were preincubated in 5% CO_2_/95% air humidified atmosphere at 37°C and followed by seeding 2000 HEK293T cells, 1000 A549 and 1000 H460 cells respectively into per well of 96-well plates. After the cells were cultured for indicated days, 10 μl CCK-8 solutions (CK04, Dojindo Molecular Laboratories) were added into each well and incubated at 37°C for 2 hours. The absorbance was measured by a Synergy H4 Hybrid Reader (BioTek) at a wavelength of 450 nm. Cell growth was assessed by cell numbers that had been calculated based on the standard curve between absorbance values and cell numbers. Each sample was triplicate.

### Statistical analysis

Student's *t*-test was used to evaluate differences between two groups. A *p* value of less than 0.05 or 0.01 was considered statistically significant.
